# The Season and Its Diseases

**Published:** 1855-09

**Authors:** 


### THE SEASON AND ITS DISEASES.

The warm season of the present year has been in every respect unlike that of 1854, which will be long remembered for its intense heat and protracted drought. The current season has been as propitious as the past was unfavorable to the growth of vegetation. From March to September everything in the weather has conspired to bring forward all the productions of the earth in the greatest profusion. The spring was dry and warm; the rains in June and August were slightly above, but in July they were below the monthly mean, and the heat has at no time been excessive. The result of this benign weather has been a most luxuriant growth of every variety of fruits and esculents, so that it may be safely said a more bountiful year was never known in Kentucky.

Until towards the latter part of July no disease could be said to prevail in Louisville. The cases of dysentery were fewer in number and of a less violent character than they have been for many summers past. Cholera has been unknown except in an occasional case imported into the city, or if an indigenous case has now and then occurred it has been at long intervals and in a way to attract no attention. Cholera morbus prevailed to some extent in June and early in July, and cholera infantum has been more general than it has been observed in this city for many years.

About the beginning of August cases of intermittent fever began to be frequent, and in the suburbs of the city and in the surrounding country this complaint has been for more than a month past nearly universal. Quinine and febrifuge compounds have been in the greatest demand. We suppose that never before in the same period were as large quantities of such medicines taken in Louisville. Concurrently with the chills and fever remittent fever has prevailed, but by no means extensively nor in a severe form. It is many years since as many people were indisposed in the city and its immediate neighborhood, but it is seldom that our bills of mortality for two months have been so small as have been those of the last two months.

We have remarked some interesting features in the prevailing fevers, the first of which is this mildness of the type. The remittent cases have been readily transformed into intermittents, and the intermittents have yielded promptly to the use of quinine. Relapses have been frequent, as they must always be when the subjects remain exposed to the action of the fever poison; but in such cases the preparations of iron have proved an effectual remedy.

We have been called to children in several instances in whom the chill was accompanied with convulsions. In one week we met with five cases of this character. The convulsions never recurred in any instance, and in all the cases but one the subsequent symptoms were mild and tractable.

In the case of a man south of Broadway, instead of chills the paroxysm was ushered in by an eruption resembling urticaria, which returned on alternate days about the same hour and receded after a few hours. He was cured by quinine, but after an interval of a week the eruption returned and required the renewed use of the fibrifuge. He has since suffered a second relapse, but this time the complaint assumed the usual form of chills and fever.

A few cases have been reported to us in which, after a number of paroxysms, the disease has terminated without treatment, but in a vast majority of the cases where medication has not been employed it has persisted, the sufferers becoming deeply anemic, with enlarged spleens, anasarca, and their usual attendants. Owing to the general prevalence of the disease among the poor, numerous opportunities have been offered for observing its tendency to a spontaneous cure. There seems to be in intermittent fever the slightest possible tendency of the sort.

The best results have been obtained in the cases of a remittent type from the constant, free application of cold water to the surface. By keeping the clothes of the patient wet, the fever has been subdued in the course of a few hours, when quinine became admissible, and recovery was prompt.

September, up to the present date (19th), has been characterized by the temperature of summer, and the rains have been frequent and copious. Still, there is rather an abatement than an increase of these fevers, and the mortality of the city was never less in the healthiest seasons.

